# Identification of candidate genes influencing anthocyanin biosynthesis during the development and ripening of red and white strawberry fruits via comparative transcriptome analysis

**DOI:** 10.7717/peerj.10739

**Published:** 2021-02-02

**Authors:** Fengli Zhao, Pan Song, Xiangfen Zhang, Gang Li, Panpan Hu, Ali Aslam, Xia Zhao, Houcheng Zhou

**Affiliations:** Zhengzhou Fruit Research Institute, Chinese Academy of Agricultural Sciences, Zhengzhou, China

**Keywords:** Strawberry, Transcriptome, Anthocyanin, Sugars, Phytohormones

## Abstract

Strawberries are one of the most economically important berry fruits worldwide and exhibit colours ranging from white to dark red, providing a rich genetic resource for strawberry quality improvement. In the present study, we conducted transcriptome analyses of three strawberry cultivars, namely, ‘Benihoppe’, ‘Xiaobai’, and ‘Snow White’, and compared their gene expression profiles. Among the high-quality sequences, 5,049 and 53,200 differentially expressed genes (DEGs) were obtained when comparing the diploid and octoploid strawberry genomes and analysed to identify anthocyanin-related candidate genes. Sixty-five DEGs in the diploid genome (transcriptome data compared to the diploid strawberry genome) and 317 DEGs in the octoploid genome (transcriptome data compared to the octoploid strawberry genome) were identified among the three cultivars. Among these DEGs, 19 and 70 anthocyanin pathway genes, six and 42 sugar pathway genes, 23 and 101 hormone pathway genes, and 17 and 104 transcription factors in the diploid and octoploid genomes, respectively, correlated positively or negatively with the anthocyanin accumulation observed among the three cultivars. Real-time qPCR analysis of nine candidate genes showed a good correlation with the transcriptome data. For example, the expression of PAL was higher in ‘Benihoppe’ and ‘Xiaobai’ than in ‘Snow White’, consistent with the RNA-seq data. Thus, the RNA-seq data and candidate DEGs identified in the present study provide a sound basis for further studies of strawberry fruit colour formation.

## Introduction

Strawberry (*Fragaria* ×* ananassa*) is an important horticultural crop that has excellent commercial and nutritional value and an attractive appearance. The colour of strawberries results from the accumulation of anthocyanins, with colourful fruit appealing to consumers because of their attractive appearance (Pillet et al., 2015). Anthocyanins are widespread water-soluble pigments that are responsible for the red, blue and purple colours of plant species and have a variety of functions, including attracting pollinators and predators, benefitting human health, enhancing resistance to stress and others (Zhang et al., 2018a). Cyanidin and pelargonidin are two major types of anthocyanins that impart fruit with bright or dark red colours. Pelargonidin accounts for >70% of total anthocyanins in strawberry fruits (Lin et al., 2018). Anthocyanins are synthesized via the flavonoid pathway via by two types of genes: structural and regulatory genes (Wang et al., 2018a). Structural genes play an important role in flavonoid and anthocyanin biosynthesis and include phenylalanine ammonialyase (*PAL*) (Olsen et al., 2008), chalcone synthase (*CHS*), chalcone isomerase (*CHI*), flavanone 3-hydroxylase (*F3H*), flavonoid 3′-hydroxylase (*F3′H*), dihydroflavonol 4-reductase (*DFR*), anthocyanidin synthase (*ANS*) (Honda et al., 2002; Salvatierra et al., 2013; Zhang et al., 2015), UDP glucose-flavonoid 3-O-glucosyl transferase (*UFGT*) (Zhao et al., 2012) and glutathione S-transferase (*GST*) (Gomez et al., 2011; Luo et al., 2018). In *Arabidopsis*, regulatory genes have also been reported to affect anthocyanin synthesis, including *MYB*, *bHLH*, *WRKY*, and *NAC* (Zhang et al., 2018a). Many structural and regulatory genes are have been well studied in strawberry (Carbone et al., 2009; Gu et al., 2015; Hawkins et al., 2016; Jia et al., 2016; Lin et al., 2018; Luo et al., 2018; Pillet et al., 2015; Salvatierra et al., 2013; Symons et al., 2012; Zhang et al., 2015; Zhao et al., 2018; Yuan et al., 2019). For example, PAL has been shown to catalyse the first step in the phenylpropanoid pathway and has functional specialization in abiotic environmental-triggered flavonoid synthesis (Olsen et al., 2008). *RAP* encodes the principal GST transporter for anthocyanin in strawberry foliage and fruit and can alter the colour of strawberry fruit (Luo et al., 2018). F3H is necessary for red fruit colour in *Fragaria vesca*, and the RNAi silencing of *F3H* led to a reduction in anthocyanin and flavonol contents (Jiang et al., 2013; Zhang et al., 2015). *F. ananassa* F3′H catalyses the first step in cyanidin derivative biosynthesis branch. Decreased *FaF3′H* gene expression blocks cyanidin 3-glucoside accumulation in red-flesh strawberries (Lin et al., 2018; Yuan et al., 2019). *FaF3′H* is rarely expressed during the strawberry fruit development period (Yuan et al., 2019; Zhang et al., 2015). Interestingly, the white colour of Chilean strawberry has been attributed to lower expression of *ANS* gene (Salvatierra et al., 2013).

Regulatory proteins can control anthocyanin biosynthesis by regulating the expression of structural genes at the transcriptional and post-transcriptional levels. Regulatory proteins are differentially modulated by sugar and hormones (Das et al., 2012; Gu et al., 2019; Jia et al., 2016; Jia et al., 2013; Symons et al., 2012). Sugars have traditionally been regarded as metabolic resources required for carbon skeleton construction and energy supply in plants (Jia et al., 2016). In recent years, numerous studies suggested that sugars may serve as essential signals that modulate anthocyanin biosynthesis (Jia et al., 2016; Jia et al., 2013; Symons et al., 2012). Sucrose is the primary carbon source for anthocyanin synthesis and determines fruit flavour and quality (Jia et al., 2016). The exogenous application of sucrose was shown to increase the expression of *DFR*, leucoanthocyanidin dioxygenase (*LDOX*), and *UFGT* by several hundred-fold. In contrast, the expression of *CHI*, *CHS*, and *C4H* (cinnamate-4-hydroxylase) decreases during anthocyanin biosynthesis (Das et al., 2012). The overexpression of the sucrose transporter *SUT1* in *F. ananassa* can enhance *PAL* and *CHS* expression, whereas *FaSUT1* RNAi led to significant inhibition of *PAL* and *CHS* expression, indicating that sucrose is involved in anthocyanin biosynthesis (Jia et al., 2013). Plant hormones such as auxin (IAA), cytokinins (CTKs), gibberellins (GA), jasmonate acid (JA), abscisic acid (ABA), and ethylene (Eth), also play a crucial role in the regulation of anthocyanin biosynthesis (Garrido-Bigotes, Figueroa & Figueroa, 2018; Gu et al., 2019; Liao et al., 2018; Symons et al., 2012). In strawberry fruits, ABA is considered to be a regulator of maturity in non-climacteric fruits with respect to the softening of fruits and anthocyanin accumulation (Liao et al., 2018). Exogenous ABA increases the anthocyanin content and activates the phenylpropanoid pathway in strawberry fruit (Garrido-Bigotes, Figueroa & Figueroa, 2018). Moreover, JA, sucrose, and IAA play different roles in strawberry fruit ripening along with ABA (Jia et al., 2016). During ripening, IAA and GA levels decrease while that of ABA level increases, resulting in fruit ripening and colour formation (Gu et al., 2019; Liao et al., 2018; Symons et al., 2012). The endogenous levels of GAs are regulated by gibberellin 2-beta-dioxygenase to catalyse ABA synthesis during early fruit development (Yamaguchi, 2008). Most hormones (IAA, GA, Eth, and JA) regulate the structural or regulatory genes of anthocyanin biosynthesis through cross-talk between their associated signal transduction pathways (Gu et al., 2019; Liao et al., 2018; Symons et al., 2012). In addition, MeJA induces the red coloration of fruit skin and promotes anthocyanin accumulation with the concomitant upregulation of the phenylpropanoid pathway-related genes (Garrido-Bigotes, Figueroa & Figueroa, 2018).

Transcription factors (TFs) are essential regulators for the expression of structural genes in the anthocyanin biosynthesis pathway, such as MYB (Zhang et al., 2015; Hawkins et al., 2016; Wang et al., 2019; Zhang et al., 2020), bHLH (Hartl et al., 2017; Schaart et al., 2013; Zhao et al., 2018), WRKY (Duan et al., 2018), and ERF (Zhang et al., 2018b). For example, MYB TFs (putative *MYB39* and *MYB86*) were shown to be downregulated in strawberries with a yellow pigment phenotype. In addition, *MYB1R* expression was shown to be upregulated in strawberries with the yellow pigment phenotype, indicating that MYB TFs repress or enhance anthocyanin accumulation in wild strawberry (Zhang et al., 2015). *F. ananassa MYB10* is specifically expressed during the early and late stages of anthocyanin biosynthesis in ripe fruit, while MYB1, functioning as a transcriptional repressor, regulates anthocyanin biosynthesis in strawberry fruit (Yuan et al., 2019). In addition, a candidate single nucleotide polymorphism (SNP) in *F*. *vesca MYB10* was identified and then functionally confirmed to be responsible for the yellow-coloured fruits in many *F*. *vesca* accessions (Hawkins et al., 2016). Sequence variations in the upstream regulatory region of *F. nilgerrensis MYB10* were shown to result in the low expression of *FnMYB10*, which is likely responsible for the white fruit phenotype of *F. nilgerrensis* (Zhang et al., 2020). Moreover, FabHLH3 (*F. ananassa* bHLH3) and FabHLH3 Δ (which encodes a putative negative regulator) interacts with MYBs to regulate proanthocyanidin biosynthesis in strawberries (Schaart et al., 2013). According to previous reports, several bHLH genes are responsive to fruit anthocyanin biosynthesis, as revealed by their expression profiles and network analysis (Zhao et al., 2018; Hartl et al., 2017). Furthermore, *WRKY41-1* has a similar role in *B. napus* to that of *WRKY41* in *A. thaliana*, which acts as a repressor to regulate anthocyanin biosynthesis when overexpressed in *A. thaliana* (Duan et al., 2018). It is important to note that the transcriptomes of ‘Benihoppe’ and ‘Xiaobai’ have been obtained in previous studies, with a mixture of ‘Benihoppe’ and ‘Xiaobai’ used in one study (Yuan et al., 2019), while in other studies, anthocyanin pathway genes were evaluated but other factors (sugar and hormones) were ignored (Lin et al., 2018).

**Figure 1:**
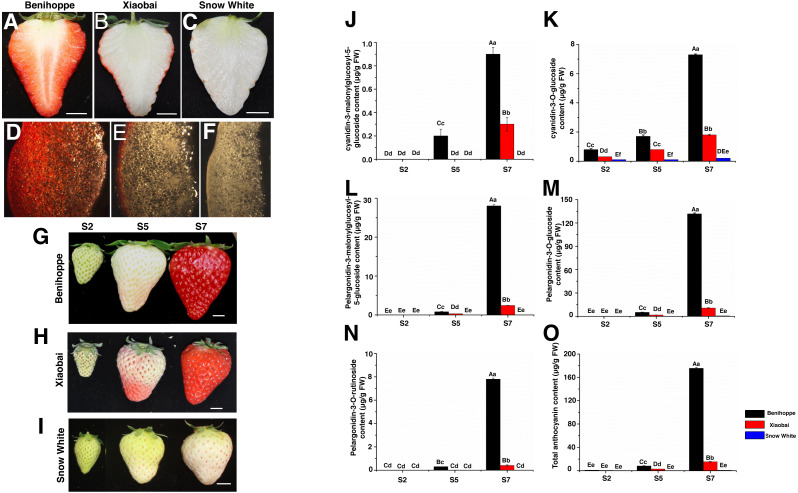
Strawberry materials used in this study. The fruit of ‘Benihoppe’(A), ‘Xiaobai’ (B), and ‘Snow White’ (C). The structure of strawberry fruit was observed by stereomicrosscope (10×) (D–F). Bar = one cm. Three fruit development and ripening stages of ‘Benihoppe’ (G), ‘Xiaobai’ (H), and ‘Snow White’ (I). Bar = one cm. (J–O) Anthocyanin content (µg g^−1^ FW) at three stages of strawberry fruit development. Values represent mean standard errors (SEs). Values followed by different letters in a column are significantly different at *P* ≤ 0.01 (capital) and *P* ≤ 0.05 (lowercase) by using SPSS software.

To elucidate the molecular mechanisms underlying the development of the white fruit flesh and skin colour of strawberries, we conducted a comparative transcriptome analysis of ‘Benihoppe’ (*F. ananassa* Duch. ‘Benihoppe’), ‘Xiaobai’ (*F. ananassa* Duch. ‘Xiaobai’), and ‘Snow White’ (*F. ananassa* ‘Snow White’). The ‘Benihoppe’ strawberry cultivar (cultivated in Japan) as red fruit skin and flesh; the ‘Benihoppe’ cultivar is a white-fleshed mutant of ‘Xiaobai’ that has red fruit skin and white flesh; and the ‘Snow White’ cultivar (cultivated in China) has white fruit skin and flesh (Figs. 1A–1F). We measured the anthocyanin and soluble sugar contents and determined the transcriptomes of these strawberry cultivars. The identification of differentially expressed genes (DEGs) and analyses of their putative biological functions and crucial pathways (anthocyanin biosynthesis and signal transduction pathway, sugar-related pathways, hormone signalling pathways, and TFs) that are predominant in strawberry cultivars with different phenotypes will enhance the current understanding of strawberry anthocyanin biosynthesis and shed light on the potential mechanism of strawberry fruit colour formation. The results of the present study will serve as a solid foundation for the future breeding of strawberry fruits.

## Materials and Methods

### Plant materials

Fruits from the three octoploid cultivated strawberry cultivars (‘Benihoppe’, ‘Xiaobai’, and ‘Snow White’) were used in the present study (Figs. 1A–1I). Plantlets of the three cultivars were grown in a greenhouse at temperatures ranging from 8 °C (dark) to 28 °C (light) and a relative humidity ranging from 55–70%. All the experiments were performed in the Zhengzhou Fruit Research Institute, Chinese Academy of Agricultural Sciences (Zhengzhou, Henan, China). The fruit development and ripening process was divided into three visual stages: S2, middle green fruit; S5, initial red fruit; S7, full red fruit (Figs. 1G–1I), as described in previous reports (Fait et al., 2008; Zhao et al., 2018). Three replicates experiments were performed, with each consisting of 30 fruits. Approximately 300 g of fruits was randomly sampled and pooled, constituting one replicate for subsequent analysis (RNA-seq, anthocyanin content, sugar content, and qRT-PCR). The sample from each stage was ground into a powder with liquid nitrogen and stored at −80 °C for later use in each experiment.

### RNA extraction, library construction and RNA sequencing

According to a previous study (Estrada-Johnson et al., 2017; Hu et al., 2018), the development and ripening process of three strawberry cultivars was divided into three key important stages (S2, S5, and S7) (Figs. 1G–1I) and were chosen to examine the content of the major anthocyanins (Figs. 1J–1O) and sugars. For transcriptome analysis, three key stages (S2, S5, and S7) from the three strawberry cultivars were chosen to study fruit coloration in the present study. Total RNA was isolated from the fruit powders using an E.Z.N.A Plant RNA Kit (R6827-01, Omega, USA) following the manufacturer’s instructions. The concentration and quality of total RNA were analysed on a NanoDrop 2000 spectrophotometer (Thermo Fisher, USA), and RNA degradation and contamination were assessed using 1% agarose gels. Two biological replicates were performed per group. For each sample, 2 µg of total RNA was used for library construction and subjected to deep sequencing on an Illumina HiSeq X Ten platform (BerryGenomics Corporation, Beijing). All sequencing data have been submitted to the NCBI Sequence Read Archive (SRA accession number PRJNA552213).

### Transcriptome and transcript analysis

Reads from each library were assembled separately. Raw sequence reads were filtered using the Illumina pipeline according to the methods of BerryGenomics, Beijing, China. Briefly, after removing the adaptor sequences and low-quality sequences (including the reads with percentages of N over 10%), the remaining high-quality clean reads were used for analysis (Tables S1–S3). The clean sequence data was compared to the *Fragaria vesca* diploid strawberry genome (National Center for Biotechnology Information (NCBI) database (https://www.ncbi.nlm.nih.gov/genome/3314), abbreviated as in the diploid genome) and the *Fragaria* x *ananassa* Camarosa genome (the genome database for rosaceae (GDR, https://www.rosaceae.org/species/fragaria_x_ananassa/genome_v1.0.a1), abbreviated as in the octoploid genome) to identify all DEGs in S2 vs. S5, S2 vs. S7, S5 vs. S7 stage comparisons for the ‘Benihoppe’, ‘Xiaobai’, and ‘Snow White’ cultivars, respectively.

**Figure 2:**
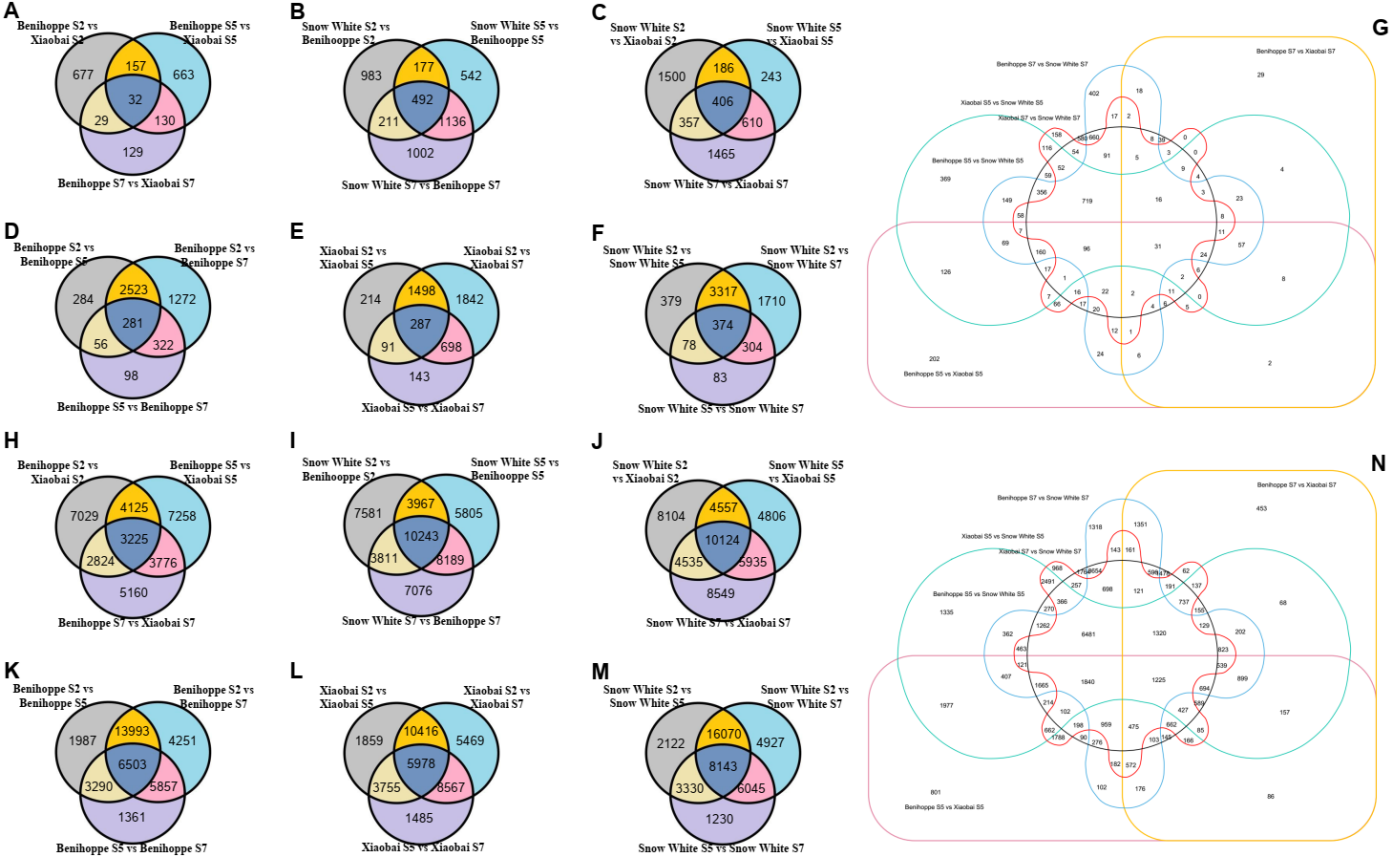
Number of differentially expressed genes among three stages of three strawberry fruit samples. Venn diagrams for the different DEGs (DEGs, Fold Change ≥ 2 and false discovery rate (FDR) < 0.01) between each combination compared with the diploid strawberry genome (Benihoppe vs. Xiaobai (A), Benihoppe vs. Snow White (B), Xiaobai vs. Snow White (C). Three stages (S2, S5, and S7) of Benihoppe (D), Xiaobai (E), Snow White (F)). Number of common and specific DEGs in six combinations compared with the diploid strawberry genome (G). Venn diagrams for the different DEGs (DEGs, Fold Change ≥ 2 and false discovery rate (FDR) < 0.01) between each combination compared with the octoploid strawberry genome (Benihoppe vs. Xiaobai (H), Benihoppe vs. Snow White (I), Xiaobai vs. Snow White (J). Three stages (S2, S5, and S7) of Benihoppe (K), Xiaobai (L), Snow White (M)). Number of common and specific DEGs in six combinations compared with the octoploid strawberry genome (N).

**Figure 3:**
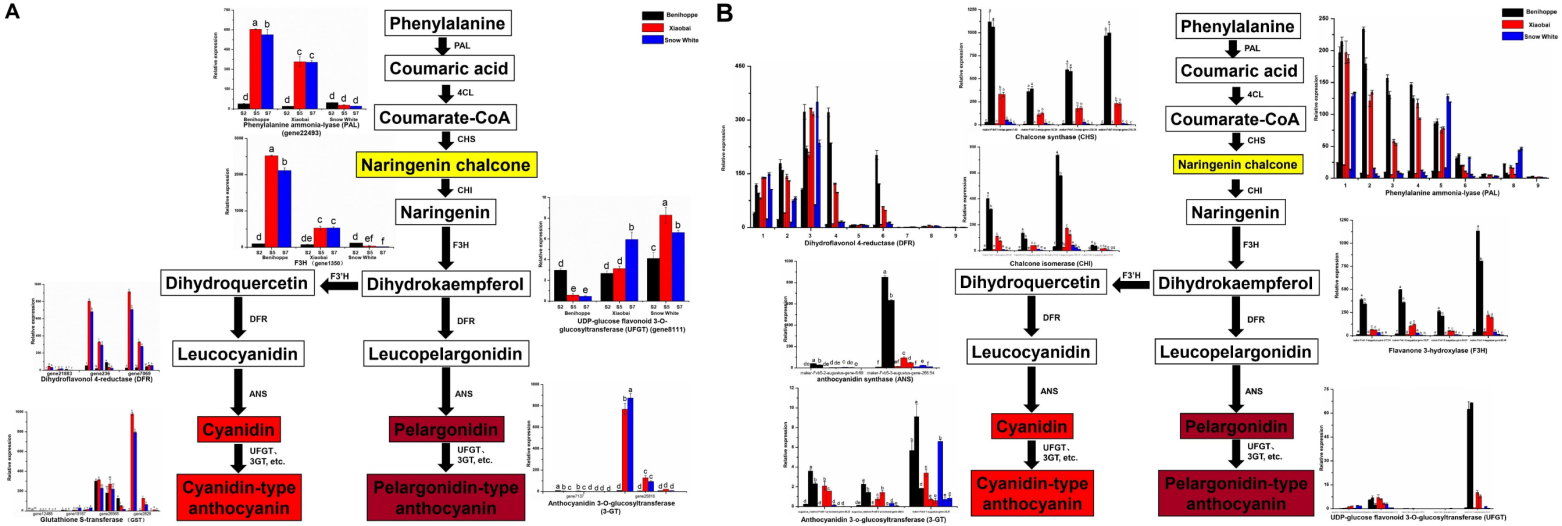
Simplified scheme of anthocyanin biosynthetic and regulatory pathway in plants. (A) DEGs of anthocyanin biosynthesis pathway genes in strawberry fruit compared with the diploid strawberry genome. *4CL* 4-coumarate CoA ligase, *F3′H* flavonoid 3′-hydroxylase. (B) DEGs of anthocyanin biosynthesis pathway genes in strawberry fruit compared with the octoploid strawberry genome. Values followed by different letters in a column are significantly different at P ≤ 0.05 (lowercase) by using SPSS software. PAL (1: maker-Fvb7-1-augustus-gene-214.51, 2: maker-Fvb6-2-augustus-gene-174.36, 3: maker-Fvb6-4-augustus-gene-113.28, 4: maker-Fvb6-1-augustus-gene-255.38, 5: maker-Fvb7-1-augustus-gene-236.35, 6: maker-Fvb7-4-augustus-gene-83.24, 7: maker-Fvb7-2-augustus-gene-144.48, 8: maker-Fvb7-3-augustus-gene-86.41, 9: maker-Fvb7-2-augustus-gene-211.53), DFR (1: augustus_masked-Fvb2-1-processed-gene-255.5, 2: maker-Fvb2-1-augustus-gene-255.45, 3: maker-Fvb2-3-augustus-gene-33.39, 4: snap_masked-Fvb2-3-processed-gene-33.26, 5: maker-Fvb2-4-snap-gene-235.71, 6: maker-Fvb2-4-snap-gene-258.87, 7: maker-Fvb3-1-augustus-gene-188.21, 8: maker-Fvb3-2-augustus-gene-132.25, 9: maker-Fvb3-4-snap-gene-175.42).

**Figure 4:**
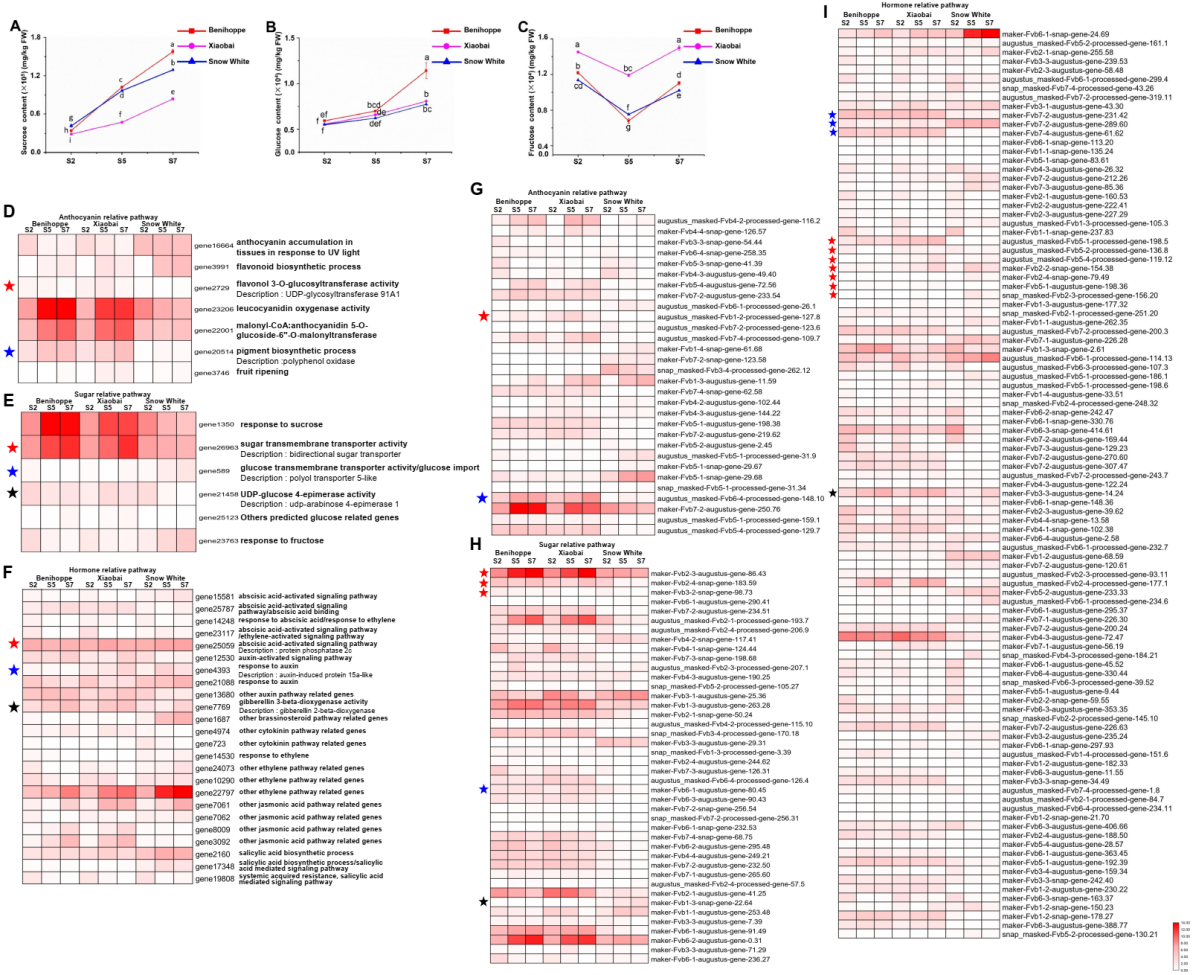
Differences in sugar content and DEGs among the three strawberry cultivars at three stages. Sugar (sucrose (A), glucose (B), fructose (C)) content of three strawberry fruit cultivars at seven developmental stages. Bars represent standard errors of the mean. Values followed by different letters in a column are significantly different at P ≤ 0.05 (lowercase) by using SPSS software. DEGs (value of log_2_(one sample/one sample) >1 or < − 1 was the selection criteria) related to anthocyanin (D), sugar (E) and hormone (F) pathway among the three strawberry cultivars at three stages compared with diploid strawberry genome. DEGs (value of log_2_(one sample/one sample) >1 or < − 1 was the selection criteria) related to anthocyanin (G), sugar (H) and hormone (I) pathway among the three strawberry cultivars at three stages compared with octoploid strawberry genome.

**Figure 5:**
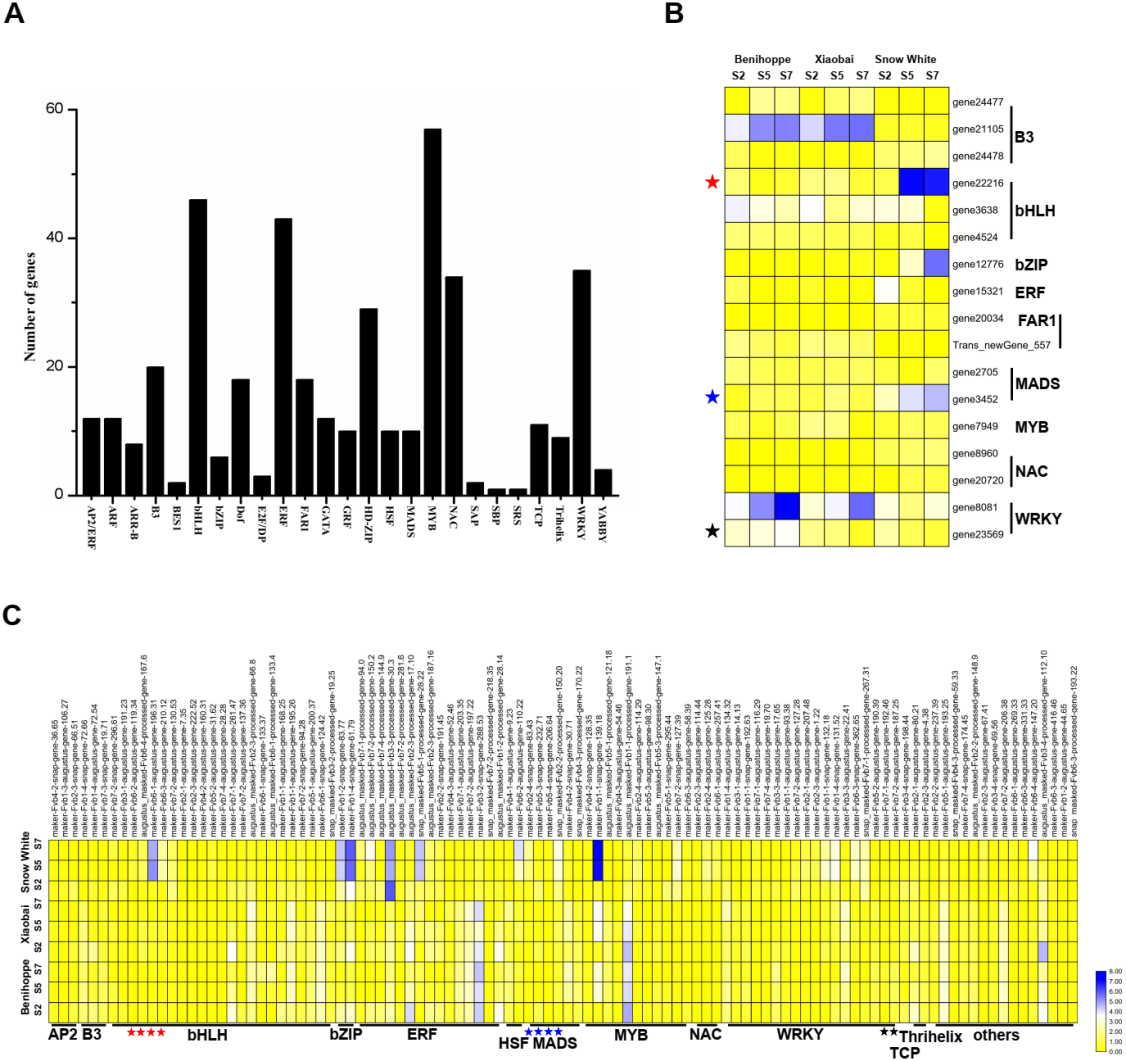
Transcription factors differentially expressed among the three strawberry cultivars at three stages. (A) Number of DEGs (DEGs, Fold Change ≥ 2 and false discovery rate (FDR) < 0.01) in different transcription factor families. (B) Heat map showing major TFs (value of log_2_(one sample/one sample) >1 or < − 1 was the selection criteria) that were differentially expressed in white and red strawberry fruits at three stages compared with diploid strawberry genome. (C) Heat map showing major TFs (value of log_2_(one sample/one sample) >1 or < − 1 was the selection criteria) that were differentially expressed in white and red strawberry fruits at three stages compared with octoploid strawberry genome.

The programs Cuffquant and Cuffnorm used fragments per kilobase of transcript per million fragments mapped (FRKM) as a measure of transcript or gene expression levels (Mortazavi et al., 2008). DEseq2 was used to identify DEGs from two samples, and the results of all statistical tests were adjusted with a fold change ≥ 2 and false discovery rate (FDR) <0.01 (Love, Huber & Anders, 2014). Venn diagrams for the different DEGs between each combination compared to the diploid strawberry genome (Benihoppe vs. Xiaobai, Benihoppe vs. Snow White, and Xiaobai vs. Snow White) (Figs. 2A–2C). Three stages (S2, S5, and S7) of Benihoppe, Xiaobai, Snow White (Figs. 2D–2F) were evaluated. A number of common and specific DEGs were identified in the six assayed combinations compared to the diploid strawberry genome (Fig. 2G). Venn diagrams were generated for the different DEGs between each combination compared to the octoploid strawberry genome (Figs. 2H–2N). The significance of DEGs was determined by determining the FDR adjusted *p*-value. A log_2_ value (one sample/one sample) of >1 or <-1 was the criterion used to select candidate genes (anthocyanin biosynthesis pathway (Fig. 3), anthocyanin, sugar and hormone -related pathways (Fig. 4) and TFs (Fig. 5) (Tables S4, S5). Genes were annotated according to BLAST search results that were compared to sequences in several databases, including the Gene Ontology (GO), Kyoto Encyclopedia of Genes and Genomes (KEGG), COG/KOG, Pfam, and NCBI non-redundant protein sequences (Nr) databases, as well as a manually annotated and reviewed section of the UniProt Knowledgebase database (Swiss-Prot). In addition, a heatmap was drawn using TBtools.

### Validation of gene expression profiles by qRT-PCR

To test the reliability of the RNA-seq data, nine candidate genes were selected for qRT-PCR expression analysis. Total RNA was extracted from three strawberry cultivars at three developmental stages (S2, S5, and S7) using an E.Z.N.A. Plant RNA Kit following the manufacturer’s instructions. The concentration and quality of RNA were analysed on a NanoDrop 1000. Approximately 1.5 µg of total RNA was used for cDNA synthesis using a PrimerScriptTM RT Reagent Kit with gDNA Eraser (TaKaRa, China). All the primers used in the qRT-PCR analysis were designed using Vector NTI (Table S6) without any interference of the conserved region, with an amplified product length of 150–300 bp. The cDNA concentration was adjusted based on the strawberry housekeeping gene FvRib413 (Meng et al., 2018; Su, Wu & Cui, 2016). For qRT-PCR, each 20 µl reaction contained 10 µl of 2 × LightCycler 480 SYBR Green I Master mix (Cat# 4887352001, Roche), 2 µl of 50 × diluted cDNA, 0.4 µl of each primer, and 7.2 µl of ddH_2_O, and the data were processed with LightCycler^®^ 480 software (Roche, China). The cycling program was as follows: 95 °C for 5 min followed by 45 cycles of 95 °C for 30 s, 60 °C for 30 s, and 72 °C for 30 s, which was followed by a melting curve analysis at 60−95 °C and obtained one melting point then subsequently used for further study. The relative expression level of each gene was calculated using the comparative 2^−ΔΔ^^*C*^^T^ method (Livak & Schmittgen, 2001). All analyses were repeated three times using biological replicates.

### Measurement of anthocyanins and soluble sugars

For anthocyanin extraction, 5 g of frozen powders of fruits from three stages (S2, S5, and S7) for the three strawberry cultivars were used in the present study (Table S7). To extract the anthocyanin mother liquid, an ultrasonic extraction process was performed for 30 min, and the mixture was heated in a boiling-water bath for 1 h. Subsequently, the mixture was filtered through 0.75-µm filter and analysed by high-performance liquid chromatography. The absorbance was measured at 530 nm, and the solvent flow rate was 0.8 ml/min. Three biological replicates were performed for each analysis.

Subsequently, 5 g of frozen powders of fruits from three stages (S2, S5, and S7) for the three strawberry cultivars were used for soluble sugar analysis (Table S8). The powder was added to deionized water, after which the volume was brought up to 50 ml, and the solution was incubated at 75 °C for 30 min. Then, the mixture was centrifuged at 12,000 rpm for 10 min and then filtered through a Dionex OnGuard II column to acquire a liquid supernatant, which was subsequently analysed by high-performance liquid chromatography. The methods used to determine the levels of anthocyanins and soluble sugars were provided by the Institute of pomology of the Chinese Academy of Agricultural Sciences. All analyses were repeated three times using biological replicates.

### Statistical analysis

**Figure 6:**
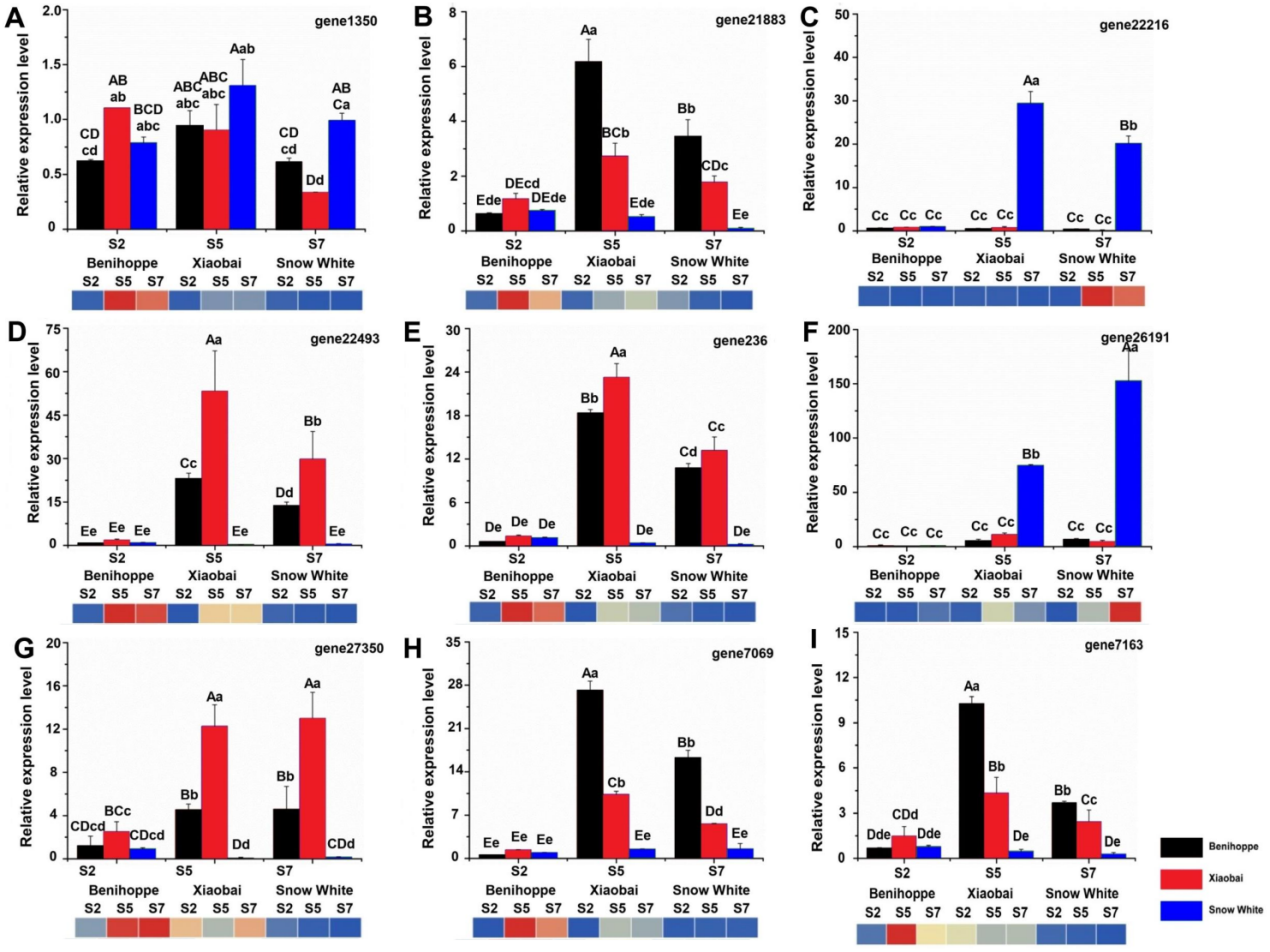
Nine genes were selected for validation of the RNA-seq data by qRT-PCR. *FvRib413* is used as an internal control. The result of qRT-PCR of FaPAL1 (D), FaF3H (A), FaDFR (B, E, H), FabHLH (C), and FaTT12 (F, G, I) expression patterns were drawn by column. The experiments were repeated three times consistent with its expression compared with the diploid strawberry genome (heatmap down line of the column). Values followed by different letters in a column are significantly different at P ≤ 0.01 (capital) and *P* ≤ 0.05 (lowercase) according to Duncan’s multiple range tests.

Each experiment was independently repeated at least three times. The data were statistically analysed with a one-way analysis of variance of Duncan’s multiple range text using IBM SPSS Statistics 20. The results are presented as the mean value ± standard deviation of the mean (SD) (Figs. 1J–1O; Fig. 3; Figs. 4A–4C; Fig. 6), and significant differences relative to controls are shown in lowercase (<0.05) and capital (<0.01) letters (Figs. 1J–1O; Fig. 3; Figs. 4A–4C; Fig. 6).

## Results

### Anthocyanin accumulation in fruits of three strawberry cultivars at different developmental stages

In the present study, the anthocyanin composition and contents of the red-fruited strawberry (‘Benihoppe’), the white-fleshed mutant of ‘Benihoppe’ (‘Xiaobai’), and the white-fruited strawberry (‘Snow White’) were analysed (Figs. 1A–1I). As expected, cyanidin (cyanidin-3-O-glucoside and cyanidin-3-malonylglucosyl-5-glucoside) and pelargonidin (pelargonidin-3-O-glucoside, pelargonidin-3-O-rutinoside, and pelargonidin-3-malonylglucosyl-5-glucoside) were the two major types of anthocyanins present in the strawberry fruits, which contributed to the red colour of the strawberry fruit (Figs. 1J–1O). Generally, the cyanidin and pelargonidin contents increased with the fruit development and ripening stages in the three cultivars. The contents of pelargonidin (167.5 mg/kg) and cyanidin (8.2 mg/kg) in ‘Benihoppe’ were significantly higher than those detected in ‘Xiaobai’ (13.3 mg/kg of pelargonidin and 2.1 mg/kg of cyanidin), while smaller amounts of cyanidin-3-O-glucoside (0.2 mg/kg) and pelargonidin-3-O-glucoside (0.7 mg/kg) were detected in ‘Snow White’ (Figs. 1J–1O, Table S7). Significant differences in the quantity of cyanidin and pelargonidin were observed between the red and white strawberry fruits, indicating the differential metabolism of anthocyanin in these cultivars.

### RNA-seq and de novo transcriptome assembly

Eighteen cDNA libraries from 3 stages (S2, S5, and S7) of ‘Benihoppe’, ‘Xiaobai’, and ‘Snow White’ with two repeats were used for sequence analysis with the Illumina HiSeq platform. These libraries were subjected to RNA-seq using an Illumina HiSeq X Ten, generating 27,673,862, 33,937,779, 27,331,497, 28,914,712, 32,588,549, 26,390,260, 25,061,869, 29,690,765, 25,703,115, 26,179,018, 26,487,231, 28,493,685, 25,410,599, 31,471,955, 30,928,839, 28,521,409, 28,248,711, and 25,042,090 150-bp paired-end raw reads (Table S1). All of the raw reads are available in the NCBI SRA database (accession number PRJNA552213). On average, 91.2% of the reads from the 18 libraries had a quality score over Q30. Moreover, over 99% and approximately 90% of the cleaned reads could be uniquely mapped to the diploid and octoploid strawberry genomes, respectively. Pearson’s correlation coefficients of the transcriptome profiles were 0.99 between each set of biological replicates, indicating that the sequencing quality was suitable for further analysis (Tables S2, S3).

### The identification of differentially expressed genes (DEGs) between genotypes and developmental stages

Using the diploid strawberry genome database, 10,354 DEGs were identified by comparing our transcriptome data with the diploid strawberry genome. A Venn diagram showing the DEGs from the three strawberry cultivars (in the diploid and octoploid genomes) was drawn using TBtools (Fig. 2). In total, 1817, 4543, and 4767 DEGs were identified for the three stages (S2, S5, and S7) in comparisons between ‘Benihoppe’ and ‘Xiaobai’ (Fig. 2A), ‘Benihoppe’ and ‘Snow White’ (Fig. 2B), and ‘Snow White’ and ‘Xiaobai’ (Fig. 2C), respectively. In addition, 4836, 4773, and 6245 DEGs were identified among the three stages (S2 vs. S5, S2 vs. S7, and S5 vs. S7) for ‘Benihoppe’ (Fig. 2D), ‘Xiaobai’ (Fig. 2E), and ‘Snow White’ (Fig. 2F), respectively. Moreover, 5049 DEGs were identified between S5 and S7 stages when comparing the Benihoppe S5 vs. Xiaobai S5, Benihoppe S5 vs. Snow White S5, Benihoppe S5 vs. Xiaobai S5, Benihoppe S7 vs. Xiaobai S7, Benihoppe S7 vs. Snow White S7, and Benihoppe S7 vs. Xiaobai S7 samples (Fig. 2G). In addition, 53,200 DEGs were obtained by comparing our transcriptome data with the octoploid strawberry genome to identify candidate genes, with 33,397, 46672, and 46,610 DEGs identified for the three stages (S2, S5, and S7) in comparisons between ‘Benihoppe’ and ‘Xiaobai’ (Fig. 2H), ‘Benihoppe’ and ‘Snow White’ (Fig. 2I), and ‘Snow White’ and ‘Xiaobai’ (Fig. 2J), respectively. Furthermore, 37,242, 37,529, and 41,867 DEGs were identified when comparing three stages (S2 vs. S5, S2 vs. S7, and S5 vs. S7) for ‘Benihoppe’ (Fig. 2K), ‘Xiaobai’ (Fig. 2L), and ‘Snow White’ (Fig. 2M), respectively. Moreover, 53,200 DEGs were identified between the S5 and S7 stages for the Benihoppe S5 vs. Xiaobai S5, Benihoppe S5 vs. Snow White S5, Benihoppe S5 vs. Xiaobai S5, Benihoppe S7 vs. Xiaobai S7, Benihoppe S7 vs. Snow White S7, and Benihoppe S7 vs. Xiaobai S7 sample comparisons (Fig. 2N). The number of DEGs identified in the octoploid genome was 10-fold higher compared to that observed in the diploid genome between the S5 and S7 stages among the three cultivars (Fig. 2). In addition, the greatest changes occurred between the S2 vs. S5, and S2 vs. S7 stages, while the smallest changes occurred between the S5 vs. S7 stages in the three octoploid strawberry cultivars. The number of DEGs in the octoploid genome was approximately 10-fold higher than that observed in the diploid genome. These results demonstrated that the genetics of ‘Benihoppe’ and ‘Xiaobai’ are similar, and relatively few DEGs were involved in the fruit ripening process compared to the assayed other combinations.

### Genes related to the anthocyanin biosynthesis pathway in the fruit development and ripening process

To investigate which anthocyanin synthesis step was blocked in ‘Xiaobai’ and ‘Snow White’, we compared the expression of anthocyanin-related genes in three developmental stages (S2, S5, and S7) of ‘Benihoppe’, ‘Xiaobai’, and ‘Snow White’ to test whether the expression of some of anthocyanin-related genes affect fruit colour. Twelve DEGs were identified in the anthocyanin biosynthesis pathway among the three cultivars in the diploid genome (as mentioned above) (Fig. 3A, Tables S4, S5), of which eleven showed significantly higher expression levels in the fruits of ‘Benihoppe’ and ‘Xiaobai’ compared to those of ‘Snow White’. In addition, only one DEG involved in the anthocyanin biosynthesis pathway was upregulated in the fruits of ‘Snow White’ compared to those of ‘Benihoppe’ and ‘Xiaobai’ (Fig. 3A, Tables S4, S5). Forty DEGs were identified in the anthocyanin biosynthesis pathway among the three cultivars in the octoploid strawberry (as mentioned above) (Fig. 3B, Tables S4, S5), of which 24 involved in the anthocyanin biosynthesis pathway were downregulated in the fruits of ‘Snow White’ compared to those of ‘Benihoppe’ and ‘Xiaobai’. Moreover, the expression levels of four structural genes in ‘Xiaobai’ were lower than these in ‘Benihoppe’ in the diploid genome, including *PAL* (gene 22493), *F3H* (gene 1350), *DFR* (gene 21883, gene 236, gene23371, and gene 7069) and *3-GT* (gene 25816 and gene 7173). One DEG (gene8111) involved in the anthocyanin biosynthesis pathway was upregulated in the fruits of ‘Snow White’ and ‘Xiaobai’ compared to those of ‘Benihoppe’, while the expression levels of these genes in ‘Snow White’ were similar to those observed in ‘Xiaobai’. In addition, the expression levels of eight structural genes in the octoploid strawberry, namely, *PAL*, *CHS*, *CHI*, *DFR*, *F3H*, *ANS*, *UFGT*, and *3-GT*, were lower in ‘Snow White’ than in ‘Benihoppe’ and ‘Xiaobai’. These results suggest the involvement of these genes in the anthocyanin biosynthesis pathway, where enhancing or repressing their expression possibly blocks anthocyanin biosynthesis in ‘Xiaobai’ and ‘Snow White’.

We also analysed seven DEGs in the diploid genome and 30 DEGs in the octoploid genome involved in anthocyanin or flavonoid biosynthesis pathways during the fruit development and ripening process among the three cultivars (Figs. 3, 4, Tables S4, S5). Among the assayed cultivars, a DEG annotated as phenylalanine ammonia-lyase had the homologous gene of gene 22493 (diploid genome) and *PAL* (nine genes in the octoploid genome chromosome 6 (three) and chromosome 7 (six)). The expression profiles showed that *PAL* (one gene in the diploid genome and five genes in the octoploid genome) was highly expressed in ‘Benihoppe’ and ‘Xiaobai’ compared to ‘Snow White’ during the fruit development and ripening process. The expression of four genes encoding PAL in the octoploid genome showed no major differences among the three cultivars (Fig. 3). Three genes in the diploid genome and nine genes in the octoploid genome (in chromosome 2 (six genes) and chromosome 3 (three genes)) were annotated as *DFR* (Fig. 3). Among them, two genes in the diploid genome and two genes in the octoploid genome were all expressed at significantly higher levels in ‘Benihoppe’ and ‘Xiaobai’ than in ‘Snow White’ during the ripening stage. The expression of the *DFR* gene located at chromosome 3 was extremely low compared to that located at chromosome 2 among the three cultivars in the octoploid genome. Gene 20514 (homologous gene augustus_masked-Fvb6-4-processed-gene-148.10 in the octoploid genome), which was predicted to encode a polyphenol oxidase gene involved in the pigment biosynthetic process, was upregulated during the fruit development and ripening process in ‘Benihoppe’ and ‘Xiaobai’ compared to that observed in ‘Snow White’ (over 50-fold higher) (Fig. 4, Tables S4, S5). Polyphenol oxidase activity is responsible for red colour and stability due to the degradation of anthocyanins, which is consistent with the anthocyanin contents observed in the three assayed cultivars (Aguilar & Hernández-Brenes, 2015). Moreover, the expression profiles of gene 2729 (homologous gene augustus_masked-Fvb1-2-processed-gene-127.8 in the octoploid genome), which is predicted to encode a UDP-glycosyltransferase 91A1 (*UGT 91A1*) involved in the flavonoid biosynthetic process, was upregulated during the fruit development and ripening process in ‘Benihoppe’ and ‘Xiaobai’ compared to that observed ‘Snow White’ (over 2-fold higher) (Fig. 4, Tables S4, S5). These results demonstrated that these genes could be involved in the anthocyanin or flavonoid biosynthetic pathways to regulate anthocyanin biosynthesis in red and white strawberry fruits by altering their transcription levels.

### DEGs involved in altering the sugar contents and sugar-related pathways in the three strawberry cultivars

To assess the association between sugar and fruit colour, we measured the contents of three sugars and analysed the expression profiles of DEGs involved in sugar-related genes according to a GO enrichment analysis (Fig. 4, Tables S4, S5). The concentration of sucrose was the highest (approximately 120 mg/g) in strawberry fruits at the S7 stage, followed by fructose (approximately 10 mg/g), and a relatively low concentration of glucose (approximately 5 mg/g) (Figs. 4A–4C, Table S8). The sucrose content was significantly different between ‘Benihoppe’ and ‘Snow White’ and was higher in these two cultivars than in ‘Xiaobai’, where the sucrose content increased with fruit development and ripening (Figs. 4A–4C, Table S8). In addition, significant differences were observed in the glucose contents of ‘Benihoppe’ compared to ‘Xiaobai’ and ‘Snow White’ at the S7 stage. It is interesting to note that the fructose content in ‘Xiaobai’ was significantly higher than that observed in ‘Benihoppe’ and ‘Snow White’ at the S2, S5, and S7 stages. The sucrose and glucose contents increased with fruit ripening stages, whereas that of fructose did not (Figs. 4A–4C). These results indicated that sugar is associated with fruit ripening.

Sugars are the primary components of fruit soluble solids that govern changes in fruit quality, which depends on starch and sucrose metabolism. Six DEGs in the diploid genome and 42 DEGs in the octoploid genome were identified as being involved in the sucrose, glucose, fructose-related pathways (Fig. 4, Tables S4, S5). Three DEGs (bidirectional sugar transporter, UDP-arabinose 4-epimerase 1, and polyol transporter 5-like (*PLT 5-like*)) have the same annotation and exhibited similar expression profiles between the octoploid and diploid genomes. It is interesting to note that the bidirectional sugar transporter is present in both the diploid (one gene, gene26963) and octoploid (three genes, maker-Fvb2-3-augustus-gene-86.43, maker-Fvb2-4-snap-gene-183.59, maker-Fvb3-2-snap-gene-98.73) genomes, and their expressions was upregulated in ‘Benihoppe’ and ‘Xiaobai’ (approximately 10-fold higher expression levels than in ‘Snow White’) compared to ‘Snow White’ (Fig. 4, Tables S4, S5). Moreover, UDP-arabinose 4-epimerase 1 expression was upregulated during the fruit development and ripening process in ‘Benihoppe’ and ‘Xiaobai’ compared to that observed in ‘Snow White’ (over 5-fold higher) (Fig. 4, Tables S4, S5). In contrast, *PLT 5-like* expression was downregulated during the fruit development and ripening process in ‘Benihoppe’ and ‘Xiaobai’ compared to that observed in ‘Snow White’ (over 3-fold higher) (Fig. 4, Tables S4, S5). The expression profiles of these DEGs were similar or opposite to the anthocyanin accumulation trend observed during the fruit development and ripening process among the three cultivars, indicating that these genes may be involved in anthocyanin biosynthesis.

### Genes involved in hormone biosynthesis or signal transduction among the three strawberry cultivars

To investigate hormone biosynthesis or pathways related to the formation of fruit colour, we analysed the expression profiles of DEGs involved in hormone-related genes according to a GO enrichment analysis (Fig. 4, Tables S4, S5). Twenty-three DEGs in the diploid genome and 101 DEGs in the octoploid genome were identified as being involved in hormone-related pathways. Auxin-induced protein 15a-like, protein phosphatase 2c 37 (*PP2C 37*), and gibberellin 2-beta-dioxygenase (*GA2Ox*) have the same annotation and expression profiles in the octoploid and diploid genomes. Among these factors, auxin-induced protein 15a-like was encoded by one gene (gene 4393) in the diploid genome and seven genes in the octoploid genome, which were located at chromosomes 2 and 5, respectively (Fig. 4, Tables S4, S5). The expression of auxin-induced protein 15a-like was higher in ‘Snow White’ than in ‘Benihoppe’ and ‘Xiaobai’ (one gene in the diploid genome and five genes in the octoploid genome). In addition, gene 25059, annotated as *PP2C 37*, was highly expressed in three varieties (insignificant difference) in the diploid genome (Fig. 4, Table S4). However, *PP2C* had three geneIDs and was located at chromosome 7 in the octoploid genome (Fig. 4, Table S5). In the octoploid genome, two *PP2C 37* genes were more highly expressed in ‘Benihoppe’ and ‘Xiaobai’ compared to ‘Snow White’ (approximately 20-fold higher). In contrast, only one gene (*PP2C 37*) was more highly expressed in ‘Snow White’ than in ‘Benihoppe’ and ‘Xiaobai’ (approximately 10-fold higher). With respect to *GA2Ox*, one gene (gene 7769 in the diploid genome) and four genes (in the octoploid genome) were upregulated during the fruit development and ripening process in ‘Benihoppe’ and ‘Xiaobai’ compared to that observed in ‘Snow White’ (two genes) (Fig. 4, Tables S4, S5). In the octoploid genome, the expression profiles of another two *GA2Ox* genes were downregulated during the development and ripening process in ‘Benihoppe’ and ‘Xiaobai’ compared to ‘Snow White’. The expression profiles of these DEGs indicated that these genes function in hormone biosynthesis or signal pathways by controlling hormone levels to regulate anthocyanin contents.

### Relationship between transcription factors and anthocyanin

To elucidate the relationship between transcription factors and the anthocyanin contents of ‘Benihoppe’, ‘Xiaobai’, and ‘Snow White’, we extracted 413 TFs from the DEGs identified at the three stages among the three cultivars and further divided them into 25 TF families according to the diploid genome (Fig. 5A). The majority of the TFs encoding DEGs were members of the MYB family, followed by the bHLH, ERF, WRKY, and NAC families (Fig. 5A, Tables S4, S5). In the present study, 17 TFs were differentially expressed in white and red fruit in the diploid genome, including the bHLH family (3 DEGs), B3 family (3 DEGs), and MADS family (2 DEGs) (Fig. 5B, Tables S4, S5). In contrast, 104 TFs were identified in the octoploid genome among the three cultivars and were further divided them into 13 TF families (contain others TFs). Most TFs belonged to bHLH family (23 DEGs), the ERF family (15 DEGs), the MYB family (11 DEGs), and the WRKY family (18 DEGs) (Fig. 5C, Tables S4, S5). Only three DEGs (*bHLH130*, *MADS23*, and *WRKY22*) were identified as having the same annotations in the diploid and octoploid genomes. The expression pattern of *bHLH130* and *MADS23* was higher in ‘Snow White’ compared to that observed in ‘Benihoppe’ and ‘Xiaobai’ and contrasted with the pattern of anthocyanin accumulation observed in the three cultivars (Tables S4, S5). However, *WRKY22* was highly expressed in ‘Benihoppe’ compared to ‘Xiaobai’ and ‘Snow White’, consistent with the accumulation of anthocyanin in strawberry fruit observed at the three stages among the three cultivars (Tables S4, S5). It is interesting to note that *MYB44* was located on three chromosomes (1, 2, and 5) and *MYB44* on chromosome 1 were more highly expressed than *MYB44* on chromosome 2 and 5. *MYB44*, which is located on chromosome 1, was upregulated in red fruit compared to white fruit. *MYB44* located on chromosomes 2 and 5 was downregulated in red fruit compared to white fruit (Table S5). In our present study, we identified many types of TFs that were differentially expressed during fruit development and ripening among the three cultivars, which suggested a strong association between these TFs and anthocyanin in strawberry.

### Validation of RNA-seq results using qRT-PCR

To validate the RNA-seq-based DEG data, we quantified the expression of nine DEGs related to anthocyanin biosynthesis using quantitative real-time PCR (qRT-PCR) for the ‘Benihoppe’, ‘Xiaobai’, and ‘Snow White’ cultivars (Figs. 6A–6I, Table S9). All selected DEGs included structural and regulatory genes involved in the anthocyanin biosynthesis pathway, such as *FaPAL1*, *FaF3H*, *FaDFR*, *FabHLH*, and *FaTT12* (*F. ananassa* transparent testa 12). In addition, the expression patterns of *FaPAL1*, *FaF3H*, *FaDFR*, *FabHLH*, and *FaTT12* were observed to be consistent with their expression profiles in diploid and octoploid genomes. The qRT-PCR results were similar to our RNA-seq results, supporting the reliability of our RNA-seq data.

## Discussion

Strawberry, an important horticultural crop worldwide, has excellent commercial and nutritional value and benefits human health (Pillet et al., 2015). Anthocyanins are the most prominent water-soluble pigments belonging to the flavonoid class (Lin et al., 2018; Zhang et al., 2015). The accumulation of anthocyanins provides strawberry cultivar fruits with different colours, ranging from white to extremely dark red. The anthocyanin content of strawberry fruit is primarily attributed to the accumulation of cyanidin (dark red colour) and pelargonidin (bright red colour), with the pelargonidin content shown to be higher than that of cyanidin in fruits (Hartl et al., 2017; Lin et al., 2018; Zhang et al., 2020). Moreover, the most predominant components of anthocyanin was identified as pelargonidin in the red flesh or skin of strawberries, while its content was extremely low in white fruit (Fig. 1, Table S7). The anthocyanin contents increase with the fruit development and ripening process (Parra-Palma, Morales-Quintana & Ramos, 2020). To identify candidate genes involved in red and white colour formation, we performed RNA-seq using three strawberry cultivars (Fig. 2, Tables S4, S5). Although previous studies obtained the transcriptome of ‘Benihoppe’ and ‘Xiaobai’, one study did not compare it with the recently drafted genome of the octoploid strawberry and a mixture of materials was used (Yuan et al., 2019), while the other study only analysed the anthocyanin pathway and transcription factors (Lin et al., 2018). Therefore, in the present study, we conducted an RNA-seq analysis of ‘Benihoppe’, ‘Xiaobai’, and ‘Snow White’ to provide valuable results to better understand strawberry fruit colour formation (Tables S2 and S3). The results showed that the number of DEGs in the octoploid genome was approximately 10-fold higher than that observed in the diploid genome, suggesting that the octoploid genome contains more replication that may be the result of an evolutionary process.

According to previous reports, genes (Supplement_sequences.txt) in the flavonoid and anthocyanin pathways, TF pathways, sugar pathways, and hormone pathways may be regulated or involved in anthocyanin biosynthesis (Das et al., 2012). Anthocyanins are the major pigments responsible for the colour of strawberry fruits (Zhang et al., 2015), the biosynthetic pathway for which is well understood and is conserved among seed plants (Yuan et al., 2019). In the present study, mixtures of the skin and flesh of strawberry fruits exhibiting different phenotypes were used to identify candidate genes by comparing RNA-seq data at three stages for the different fruits. Nineteen DEGs in the diploid genome and 70 DEGs in the octoploid genome were observed to be involved in anthocyanin biosynthesis or signalling pathways according to the annotation results (Figs. 3, 4). Nine DEGs exhibited the same annotation between the diploid and octoploid genomes that may be associated with anthocyanin biosynthesis among the white and red strawberry cultivars. These expression profiles were similar or contrasted with the observed anthocyanin content among the three cultivars of fruit, indicating that these genes possibly enhance or repress anthocyanin biosynthesis in fruit. In the present study, anthocyanin biosynthesis transcripts, including *CHS*, *CHI*, and *ANS*, which were not identified in the diploid genome but were present in the octoploid genome, were downregulated in white strawberry fruits, indicating that the transcript abundance of these genes was positively related to the accumulation of anthocyanin (Zhang et al., 2015; Parra-Palma, Morales-Quintana & Ramos, 2020). Moreover, three structural genes with the same annotation were selected from the diploid and octoploid genomes, namely, *PAL*, *DFR*, and *UFGT3* (Tables S4, S5). These structural genes shared similar expression trends in two genomes and were consistent with previous results showing higher expression of these genes (Tables S4, S5) (Yuan et al., 2019). For example, regarding *DFR*, an increasing trend in gene expression was observed during the red strawberry fruit ripening process in the diploid genome. In the octoploid genome, some copies of the *DFR* gene were highly expressed (the expression value was more than 100 fpkm), while some copies were expressed at low levels (the expression value was less than 2 fpkm). The expression profiles of *DFR* were consistent with a reduction in anthocyanin accumulation and a yellow pigment phenotype (Zhang et al., 2015). In addition, *F3′H* was not identified in the present study (no significant difference between two genomes), with similar results having been obtained in previous reports (Lin et al., 2018; Zhang et al., 2015). Furthermore, *UFGT 3* (gene 8111, diploid genome; augustus_masked-Fvb6-3-processed-gene-86.10, octoploid genome) was downregulated during the strawberry fruit ripening process, the expression of which contrasted with that of UFGT, which enhances cyanidin glucoside accumulation (Li et al., 2016). UFGT catalyses the glucosylation of both ABA and IAA in vitro and accelerates fruit ripening by decreasing ABA levels and inhibiting the early release of ethylene (Sun et al., 2017). The results indicated that genes in the anthocyanin pathway may play an important role in fruit colour formation.

How sugars and phytohormones affect anthocyanin accumulation in plants has been previously reported, providing a valuable reference for future studies conducted in other species (Das et al., 2012; Garrido-Bigotes, Figueroa & Figueroa, 2018; Gu et al., 2019; Symons et al., 2012). Sugars (sucrose, glucose, and fructose) play a role in modulating the anthocyanin synthesis pathway (Solfanelli et al., 2006). In the present study, sucrose and glucose contents increased with the fruit ripening stages in the three cultivated strawberries, while fructose levels in ‘Xiaobai’ were significantly higher than that observed in the other cultivars (Fig. 4A). Previous studies elucidated the trends in the hormone contents during the strawberry fruit development and ripening process (Garrido-Bigotes, Figueroa & Figueroa, 2018; Gu et al., 2019; Liao et al., 2018; Symons et al., 2012). In the present study, six DEGs in the diploid genome and 42 DEGs in the octoploid genome involved in sugar-related pathways were identified (Fig. 4, Tables S4, S5). Subsequently, twenty-three DEGs in the diploid genome and 101 DEGs in the octoploid genome were shown to be involved in hormone-related pathways (Fig. 4, Tables S4, S5). Eventually, six DEGs with the same annotation were identified between the diploid and octoploid genomes and shown to belong to sugar (three DEGs) and hormone (three DEGs) pathways. ABA and sucrose signalling pathways have been studied in detail in many plants (Das et al., 2012; Jia et al., 2013; Jia et al., 2016; Meng et al., 2018). Anthocyanin accumulation specifically depends on sucrose signalling (Meng et al., 2018). Moreover, exogenous sucrose application was shown to promote the maturity of strawberry fruit, which was achieved by regulating ABA levels in fruit and could dramatically accelerate fruit ripening (Jia et al., 2013). Mutant *Arabidopsi* s lines with increased anthocyanin accumulation may predominantly result from a high endogenous sucrose concentration (Meng et al., 2018). In addition, polyol transporter is a plasma membrane broad-spectrum sugar-proton symporter that mediates the uptake of linear polyols and transports different types of sugar (Klepek et al., 2005). PLT 5-like, PP2C 37, and auxin-induced protein 15a-like may negatively regulate anthocyanin, while UDP-arabinose 4-epimerase 1, bidirectional sugar transporter, and GA2Ox may positively regulate anthocyanin (Tables S4, S5). PYR is an ABA receptor that suppressed by the phosphatase activity of PP2C and causes the induction of ABA signal transduction (Klingler, Batelli & Zhu, 2010). The level of *PP2C* expression decreased with the fruit development and ripening process. The lower expression of *PP2C* can enhance the signal transduction of ABA and promote fruit ripening or anthocyanin biosynthesis. Moreover, ABA plays an important role during fruit pigmentation (Hu et al., 2019). The expression patterns of *PP2C* 37 (downregulated in the diploid genome, with two transcripts upregulated and one transcript downregulated in the octoploid genome) in our study indicated that this gene is involved in anthocyanin biosynthesis (Fig. 4, Tables S4, S5). In addition, the level of *GA2Ox* expression was increased as the strawberry fruit development and ripening process progressed. The degradation of GAs is catalysed by the *GA2Ox* enzyme (Yamaguchi, 2008). At the onset of fruit ripening, both auxin and GA levels decreased, leading to a steep increase in the endogenous level of ABA that drives fruit ripening and colour formation (Liao et al., 2018). These results demonstrated that *GA2Ox* can degrade GA to increase the ABA content and promote fruit colour formation, showing that these hormone pathway-associated DEGs are involved in anthocyanin biosynthesis and regulate fruit colour formation.

TFs are positive regulators that enhance the expression of structural genes involved in the anthocyanin biosynthesis pathway (Duan et al., 2018; Hui et al., 2019; Wang et al., 2018a; Wang et al., 2018b; Zhang et al., 2018b). Seventeen TFs (in the diploid genome) and 104 TFs (in the octoploid genome) were differentially expressed between the red- and white-fleshed strawberries. Moreover, three TFs have the same annotation in the diploid and octoploid genomes and may be associated with anthocyanin biosynthesis in the white and red strawberry cultivars. In strawberries, *bHLH* (*FabHLH3* and *FabHLH33*) has been shown to play important roles by interacting with MYB during proanthocyanidin and anthocyanin biosynthesis (Schaart et al., 2013). A previous study showed that *bHLH93* and *bHLH122* are differentially expressed between red- and white-fleshed strawberries (Lin et al., 2018). In the present study, *bHLH130* (gene 22216) was upregulated in white fruit compared to red fruit and may be negatively regulated anthocyanin biosynthesis by competitively consuming substance of anthocyanin. In addition, some *bHLHs* have also been shown to be responsible for fruit anthocyanin biosynthesis according to their expression profiles in three octoploid strawberry cultivars (Zhao et al., 2018). FaMYB44 can interact with FabHLH3 and FaTTG1 to form FaMYB44-related MBW complexes. In addition, MYB44 can be inhibited by MYB10 (induced anthocyanin accumulation) to negatively regulate sucrose accumulation (Wei et al., 2018). These results suggest that MYB44 may inhibit anthocyanin biosynthesis by regulating bHLH3 and MYB10 expression. The expression patterns of *bHLH130* and *MADS23* contrasted with the anthocyanin contents observed in the three cultivars at the three evaluated stages (Fig. 5). *VmTDR4* (MADS-box genes) plays a crucial role in controlling anthocyanin biosynthesis by acting directly or indirectly through MYB transcription factors to control carbon flux through the phenylpropanoid pathway (Jaakola, Poole & Jones, 2010). However, the pattern of *WRKY22* expression was consistent with the observed accumulation of anthocyanin in fruit detected during the three ripening stages in the three evaluated cultivars (Fig. 5). *AtWRKY41* controls the expression of three regulatory genes (*AtMYB75*, *AtMYB111*, and *AtMYBD*) and two structural genes (*AT1G68440* and *AtGSTF12*) involved in anthocyanin biosynthesis (Duan et al., 2018). Furthermore, qRT-PCR analysis showed that the expression levels of nine anthocyanin biosynthesis structural genes and regulatory genes were consistent with our RNA-seq data (Fig. 6, Table S9). For example, the expression of *PAL 1* was higher in ‘Benihoppe’ and ‘Xiaobai’ compared to that observed in ‘Snow White’ as assessed by qRT-PCR, and the qRT-PCR results were consistent with the expression of this gene detected by RNA-seq in the diploid and octoploid genomes. Taken together, the DEGs identified in the present study may explain why strawberry fruit skin or flesh can exhibit a white colour. However, additional studies are needed to verify whether these candidate genes are responsible for anthocyanin biosynthesis in strawberries.

In summary, in the present study, we measured the contents of anthocyanin and soluble sugars and performed a transcriptome analysis to assess fruit development and ripening in white- and red-skinned or fleshed strawberry cultivars. The RNA-seq results revealed a set of candidate genes (65 DEGs in the diploid genome and 317 DEGs in the octoploid genome, with 18 DEGs (in the diploid genome) and 53 DEGs (in the octoploid genome) having the same annotation between the diploid and octoploid genomes) that may be associated with anthocyanin biosynthesis in white and red strawberry cultivars. Overall, the results of the present study provide insights into the possible molecular mechanisms influencing strawberry fruit colour, which is of great importance for both basic research and the breeding of strawberry for white fruit.

##  Supplemental Information

10.7717/peerj.10739/supp-1 Supplemental Information 1 Supplemental tables. Table S1. The qulity of transcriptome sequencing data statistics.
Table S2. Pearson’s correlation coefficients between biological replicates. Of the transcriptome profiles obtained by comparing with *Fragaria vesca*.
Table S3. Pearson’s correlation coefficients between biological replicates. Of the transcriptome profiles obtained by comparing with *Fragaria x ananassa*.
Table S4. Expression patterns of candicate genes in *Fragaria vesca*. Differentially expressed genes (value of log_2_(one sample / one sample) >1 or < − 1 was the selection criteria) of the anthocyanin-related pathway, sugar-related pathway, hormonal pathways and transcription factors. The number indicated candidate gene FPKM.
Table S5. Expression patterns of candicate genes in *Fragaria x ananassa*. Differentially expressed genes (value of log_2_(one sample / one sample) >1 or < − 1 was the selection criteria) of the anthocyanin-related pathway, sugar-related pathway, hormonal pathways and transcription factors. The number indicated candidate gene FPKM.
Table S6. The primers names and sequences used in this study.
Table S7. The content of anthocyanins in three cultivated strawberry. The number indicated anthocyanins content (µg*g*^−1^ FW).
Table S8. The content of sugars in three cultivated strawberry. The number indicated sugars content (mg kg^−1^ FW).
Table S9. Expression levels of anthocyanin pathway genes by qRT-PCR. The number indicated candidate gene expression levels.

10.7717/peerj.10739/supp-2 Supplemental Information 2 Supplement sequences.

## Additional Information and Declarations

### Data Availability

The following information was supplied regarding data availability: The raw sequence reads are available at the NCBI SRA database: PRJNA552213.
